# Analysis of Passive Mixing in a Serpentine Microchannel with Sinusoidal Side Walls

**DOI:** 10.3390/mi9010008

**Published:** 2017-12-28

**Authors:** Muhammad Usman Javaid, Taqi Ahmad Cheema, Cheol Woo Park

**Affiliations:** 1 School of Mechanical Engineering, Kyungpook National University, 80 Daehak-ro, Bukgu, Daegu 41566, Korea; usmanjavaid90@gmail.com; 2 Department of Mechanical Engineering, Ghulam Ishaq Khan Institute of Engineering Sciences and Technology, Topi 23460, Khyber Pakhtoon Khwa, Pakistan; tacheema@giki.edu.pk

**Keywords:** micromixer, chaotic advection, serpentine-shaped microchannel, mixing index

## Abstract

Sample mixing is difficult in microfluidic devices because of laminar flow. Micromixers are designed to ensure the optimal use of miniaturized devices. The present study aims to design a chaotic-advection-based passive micromixer with enhanced mixing efficiency. A serpentine-shaped microchannel with sinusoidal side walls was designed, and three cases, with amplitude to wavelength (*A*/*λ*) ratios of 0.1, 0.15, and 0.2 were investigated. Numerical simulations were conducted using the Navier–Stokes equations, to determine the flow field. The flow was then coupled with the convection–diffusion equation to obtain the species concentration distribution. The mixing performance of sinusoidal walled channels was compared with that of a simple serpentine channel for Reynolds numbers ranging from 0.1 to 50. Secondary flows were observed at high Reynolds numbers that mixed the fluid streams. These flows were dominant in the proposed sinusoidal walled channels, thereby showing better mixing performance than the simple serpentine channel at similar or less mixing cost. Higher mixing efficiency was obtained by increasing the *A*/*λ* ratio.

## 1. Introduction

Microfluidic devices use fluid flow at the submillimeter scale for applications in areas such as life sciences, analytical chemistry, and bioengineering. Small sample volume consumption, low cost, flexible and controlled operation, and high throughput make the use of microfluidic devices desirable [1]. Micro total analysis systems and microscale devices, which are employed for biochemical analyses and processes, such as protein folding, enzyme reactions, and drug delivery, require rapid mixing of reagents before a chemical reaction could occur [2]. Given the small characteristic dimensions at the microscale, the Reynolds number is low and the flow is laminar. Thus, without turbulent mixing, only molecular diffusion causes mixing, but is a slow process [3]. Therefore efficient mixing mechanisms are necessary to achieve the realized potential of lab-on-a-chip technologies.

Almost three decades ago, Ottino presented an overview of earlier work on mixing and chaotic advection, and comprehensive theory on kinematics and chaotic dynamics [4,5]. Following the interest of researchers in microfluidics, Ottino and Wiggins provided a review of mixing at microscale, and the mathematical foundations of chaotic mixing for design of efficient micromixers [6,7]. Past researchers have developed active and passive techniques to attain rapid mixing by influencing the flow to cause chaotic advection or to increase the contact area of fluid layers [8]. Active micromixers use external energy sources, such as pressure [9], acoustic [10,11,12], and electric field [13]. Active mixers such as the acoustic-based mixers with sharp edges [10,11,12] have been shown to achieve higher mixing efficiency than passive mixers, but they need an external energy source. By contrast, passive mixers use geometric characteristics to split, stretch, fold, and break the fluid streams, thereby enhancing mixing [14]. Although the fabrication of passive mixers is complex, the absence of an external driver and the ease of integration in microsystems provides them an edge over their active counterparts [8].

Previous studies on passive mixers have mainly relied on lamination- and chaotic advection-based designs to enhance mixing [14]. Buchegger et al. reported a multi-lamination mixer that used wedge-shaped vertical inlets, leading to a single horizontal channel, where efficient mixing of four streams occured [15]. Nimafar et al. proposed a mixer with H-shaped channels, for the splitting and recombination of two fluids. Experimental comparisons with T- and O-micromixers showed the superior performance of their device [16]. An experimental and numerical study on a crossing manifold micromixer has shown that the change in flow profile due to non-uniform momentum, along with convection and increasing interfacial area, improved mixing efficiency [17]. A three-layer split and recombination mixer achieved a high mixing efficiency because the design allowed for three times the surface area than a simple T-mixer could provide for diffusion to occur [18]. Kim et al. used chaotic advection and splitting and recombination in their serpentine laminating mixer. Three-dimensional serpentine mixing units produced chaotic advection, and F-shaped mixing units caused splitting and recombination [19].

Other strategies to enhance passive mixing utilize chaotic advection induced by geometric manipulations. Mengeaud et al. conducted an optical and numerical investigation on a zigzag microchannel. Molecular diffusion was the dominant factor for mixing at Reynolds numbers lower than 80, and secondary flows affected species mixing at high Reynolds numbers [20]. Hossain et al. used numerical investigation to compare the mixing performance of zigzag, curved, and square-wave-shaped channels and concluded that the square-wave-shaped channel exhibited better performance than the other two geometries [21]. Parsa and Hormozi investigated mixing in sinusoidal channels by varying the phase shift between side walls. High mixing indices were achieved for phase shifts of *π*/2 and 3*π*/2 [22]. In another study, Parsa et al. investigated the effect of the amplitude to wavelength (*A*/*λ*) ratio of sinusoidal walls and observed the best performance at high *A*/*λ* ratios [23]. Afzal and Kim used coupling of pulsatile flow and sinusoidal walled convergent–divergent channel to achieve high mixing efficiency [24]. Asymmetric curvilinear microchannels can show better mixing performance than symmetric channels at Dean numbers (*K*) greater than 16.8, whereas symmetric channels achieve a higher mixing index than asymmetric curvilinear microchannel below the threshold value of *K* [25]. Fan et al. presented a study on the use of sharp corners in series to improve mixing efficiency [26]. Alam et al. presented a numerical study of straight and curved microchannels with circular obstacles that changed the flow pattern to achieve better performance than channels without obstacles [27].

Apart from achieving high mixing efficiency, micromixers should also process samples without damaging large biomolecules. Obstacle-based passive designs have high local strains and can damage biomolecules due to shear. Serpentine-shaped mixers can prevent this damage because, chaotic advection due to high local strains does not occur in these channels [28]. Therefore serpentine channels can be employed to achieve high mixing efficiency and damage-free processing of samples. Modifications, such as the use of non-aligned inputs [29] and three-dimensional serpentine geometries have been shown to further enhance the mixing performance of serpentine channels [28]. The study conducted by Alam and Kim showed that modifying the side walls of curved serpentine channels can also improve mixing performance [30]. They used rectangular grooves at specified locations and observed increased mixing as groove width expanded. Meanwhile groove depth only slightly affected the mixing index.

This paper reports the results of a numerical study on the mixing performance of a chaotic-advection-based serpentine microchannel with sinusoidal side walls. Sinusoidal walled channels have been shown to achieve better mixing performance than straight channels [22,23], however, the geometries used in the previous studies on sinusoidal walled channels were made by the modification of a straight channel. The present study presents a modified geometry that was designed using sinusoidal side walls in a serpentine channel to increase the secondary flow because of change in Dean number, and attain better mixing than a simple serpentine channel. The mixing performances of a simple serpentine channel and a serpentine channel with sinusoidal walls, with Reynolds numbers ranging from 0.1 to 50, were compared. A simple serpentine geometry similar to the square wave channel [21] and serpentine microchannel [29] reported by Hossain et al. was chosen to compare the mixing performance of proposed micromixer. Two additional cases of sinusoidal walled serpentine channel were considered by increasing the (*A*/*λ*) ratio of sinusoids. Micromixers with sinusoidal walls showed better mixing efficiency than a simple serpentine channel, and the increase in *A*/*λ* ratio further enhanced the performance.

## 2. Micromixer Design

**Figure 1:**
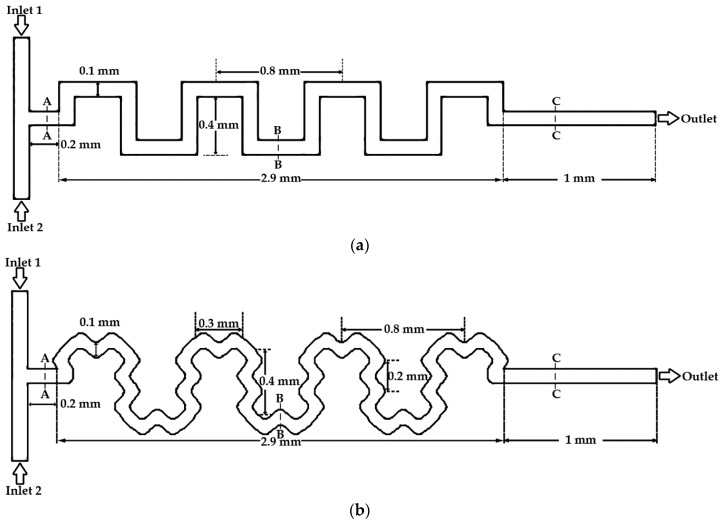
Schematics of microchannels. (**a**) Simple serpentine channel and (**b**) serpentine channel with sinusoidal walls.

The simple serpentine channel and serpentine channel with sinusoidal walls used in the present study are shown in Figure 1a,b, respectively. Both designs used two inlets, connected by a T-joint, that lead the fluids into the mixing channel. Before entering the serpentine-shaped part, fluids passed through a straight channel of 0.2 mm in length for both geometries. The inlets have a square cross section, and both planar devices have a width and depth of 0.1 mm. The sinusoidal walls shown in Figure 1b were generated using the following function:

(1) $$y=A\sin\left(\frac{2\pi x}{\lambda }\right)$$

where *A* is the amplitude and *λ* is the wavelength of the sinusoids. Three cases of serpentine channels with sinusoidal walls with amplitudes of 0.02, 0.03, and 0.04 mm corresponding to *A*/*λ* ratios of 0.1, 0.15, and 0.2, respectively, were considered in this study. For the serpentine channel with sinusoidal walls, the sinusoids at the outer turns were joined using quadratic curves. The dimensions for both geometries used in the present study are shown in Figure 1.

## 3. Numerical Model

The single-phase, incompressible, and steady-state laminar flow in micromixers was solved for momentum and mass conservation using the Navier–Stokes equations and the continuity equation, respectively. The equations are expressed as follows:

(2) $$\rho \left(u\cdot \nabla \right)u=\nabla \cdot \left[-pI+µ\left(\nabla u+{\left(\nabla u\right)}^{T}\right)\right]$$

(3) ρ∇·(u)=0

Equation (2) represents momentum conservation in which *ρ* is the density of fluid (kg·m^−3^), ***u*** is the velocity vector (m·s^−1^) *p* is the pressure (Pa), ***I*** is the unit diagonal matrix, and *µ* is the dynamic viscosity of the fluid (kg·m^−1^·s^−1^). Equation (3) is the continuity equation. The solution of these equations yielded the velocity and pressure fields. The obtained velocity field was used to compute species concentration field using the convection–diffusion equation expressed as follows:

(4) ∇·(−D∇c)+u·∇c=R

where *D* is the diffusion coefficient (m^2^·s^−1^), *c* is the species concentration (mol·m^−3^), and *R* is the reaction rate, which was assumed to be zero in this case.

## 4. Mixing Analysis

Methods based on striation thickness of fluid layers [31] and standard deviation of concentration [32] have been used in the past to characterize the mixing performance of micromixers. The formula employed in the present study to calculate mixing index (MI), based on the standard deviation of concentration is expressed as follows:

(5) $$\mathrm{MI}=1-\frac{\sigma }{\sigma_{\mathrm{Max}}}$$

where *σ* is the standard deviation of species concentration in any given cross section and *σ*_Max_ is the standard deviation of the completely unmixed state. The value of the mixing index is 0 and 1 for the unmixed and fully mixed states, respectively. The standard deviation is expressed as follows:

(6) $$\sigma =\sqrt{\frac{1}{N}\sum_{i=1}^{N}{\left(c_{i}-c_{m}\right)}^{2}}$$

where *N* is the number of sampling points, *c_i_* is the mixing fraction at point *i*, and *c_m_* is the optimal mixing fraction.

The power required to drive fluids through the channel should also be taken into account while characterizing the mixing performance of any design. Usually high mixing efficiency at increased flow rates results in higher power consumption [33]. So the cost of mixing should also be determined. This mixing cost is calculated in terms of pressure drop using the mixing index to pressure drop (MI/Δ*P*) ratio [34], or in terms of input power [35]. The formula used to calculate mixing cost (MC) in terms of input power is expressed as follows:

(7) $$\mathrm{MC}=\frac{Input\;Power}{\mathrm{MI}}=\frac{\Delta P\cdot Q}{\mathrm{MI}}$$

where Δ*P* is the pressure drop (Pa) across the channel and *Q* is the corresponding flow rate (m^3^·s^−1^).

## 5. Model Implementation

Water and a dilute dye solution were considered as working fluids, each with a density of 1000 kg·m^−3^ and a dynamic viscosity of 0.001 kg·m^−1^·s^−1^. The diffusion coefficient of 10^−10^ m^2^·s^−1^ was used for the dye solution in water. Any change in the physical properties of the fluid due to the presence of solute was ignored. To solve fluid flow, the no-slip boundary condition was set at the walls along with zero pressure at the outlet, and symmetry in the vertical direction. Velocity was used at the inlets, and simulations were conducted at various Reynolds numbers ranging from 0.1 to 50. Reynolds number is defined as follows:

(8) $$Re=\frac{\rho UD_{h}}{µ}$$

where *Re* is the Reynolds number, *ρ* is the fluid density (kg·m^−3^), *U* is the fluid velocity (m·s^−1^), *D_h_* is the hydraulic diameter (m), and *µ* is the dynamic viscosity (kg·m^−1^·s^−1^) of the fluid. Inlet concentrations of 0 and 1 were used at the two inlets to compute the concentration field.

**Figure 2:**
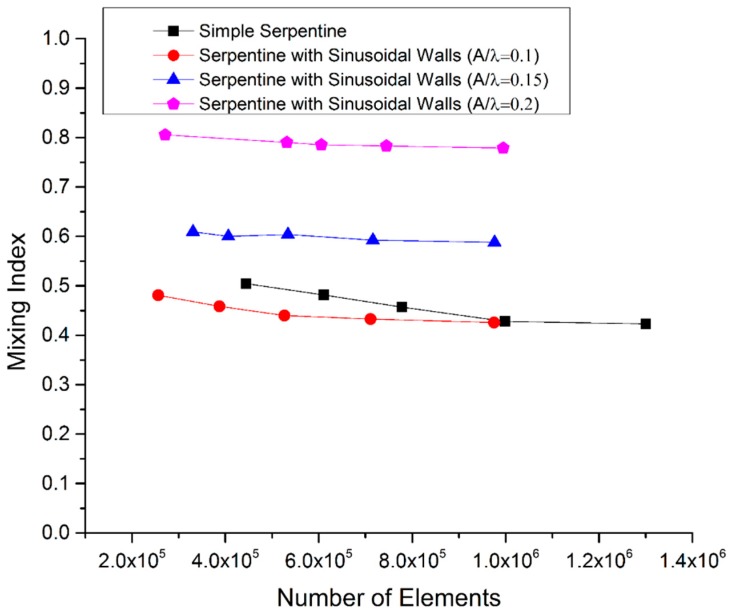
Mesh independence test at Reynolds number (*Re*) = 40.

COMSOL Multiphysics (Version 5.3, COMSOL Inc., Burlington, MA, USA) was used for the simulations using the laminar flow and transport of diluted species interfaces. The domain was discretized using tetrahedral elements. Grid independence tests were carried out with different numbers of mesh elements (Figure 2). Finally to save the computational cost, 990,338 elements were used for the simple serpentine geometry. For three different cases of serpentine channels with sinusoidal walls, 710,957, 715,895, and 744,686 elements were used for the geometries with *A*/*λ* ratios of 0.1, 0.15, and 0.2, respectively. In numerical simulations, the discretization of convective terms for determining the concentration distribution causes numerical errors that result in the addition of numerical diffusion. The extent of numerical diffusion can be minimized using higher order discretization [36]. To reduce the extent of artificial diffusion, a higher order discretization was used in the present study. All simulations were conducted on a Windows 7 operated workstation with an Intel Xeon E5-2620 v3 2.4 GHz processor (Intel Corporation, Santa Clara, CA, USA) and 32 GB random access memory (RAM).

## 6. Results and Discussion

**Figure 3:**
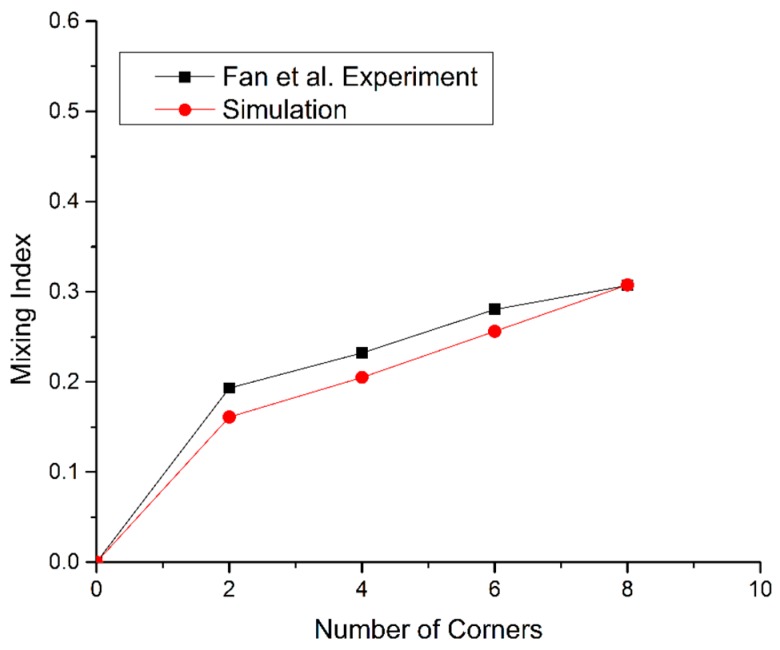
Comparison of the simulation and experimental results.

The numerical model was validated by comparing its results with experimental results of Fan et al. [26]. Simulations were conducted using the reported geometric parameters and fluid properties. The results shown in Figure 3 validate the numerical model by showing close agreement of the simulation results with the experimental results.

Four cases, namely, simple serpentine channel (Case 1), serpentine channel with sinusoidal walls and *A*/*λ* ratio of 0.1 (Case 2), serpentine channel with sinusoidal walls and *A*/*λ* ratio of 0.15 (Case 3), and serpentine channel with sinusoidal walls and *A*/*λ* ratio of 0.2 (Case 4), were considered in the present study. In curvilinear channels, the fluid motion towards the outer wall due to centrifugal forces creates velocity and pressure gradients that cause vortical flows. The magnitude of these secondary flows increases with the increase in Dean number (*K*) which is defined as follows:

(9) $$K=\sqrt{\frac{D_{h}}{2r}}Re$$

where *D_h_* is the hydraulic diameter (m), *Re* is the Reynolds number, and *r* is the radius of curvature (m). The serpentine shape considered in the present study has sinusoidal walls as well as turns of the serpentine. Determination of radius of curvature and calculation of Dean number is difficult because of this geometric complexity. Equation (9) shows that for a geometry with fixed hydraulic diameter, the Dean number can be increased by increasing the Reynolds number or decreasing the radius. From this we can say that the Dean number in sinusoidal walled channels with a higher *A/λ* ratio would be greater because of increased curvature.

**Figure 4:**
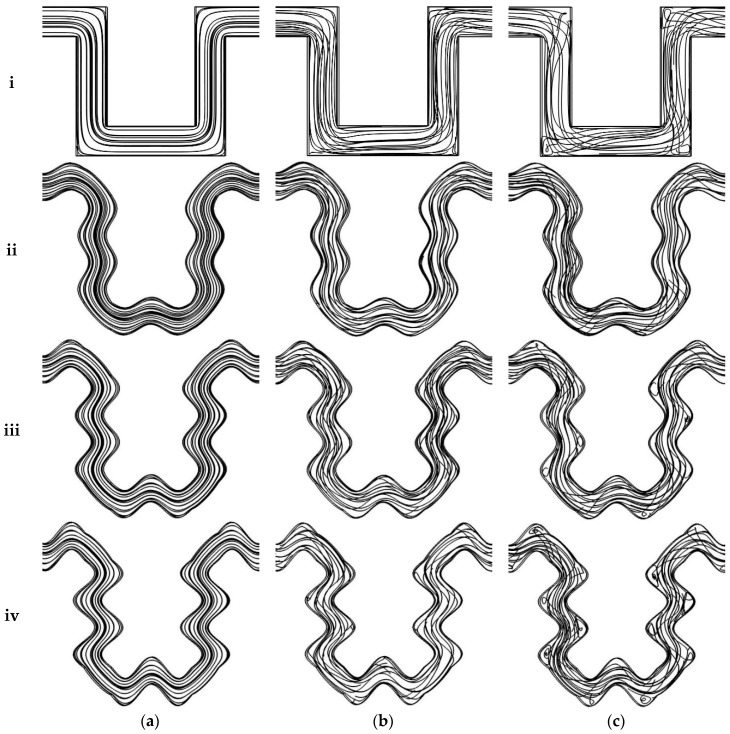
Streamline plots in first U-shaped region of all geometries: (**i**) Case 1; (**ii**) Case 2; (**iii**) Case 3; and (**iv**) Case 4 at (**a**) *Re* = 0.1; (**b**) *Re* = 20; and (**c**) *Re* = 50.

Streamline plots at Reynolds numbers of 0.1, 20, and 50 are shown in Figure 4 to understand the effect of secondary flows and the mixing phenomenon at different flow rates. An increase in Reynolds number will cause an increase in Dean number and as a result a change in streamlines trajectories for all cases can be seen because of increased secondary flow. At Reynolds number of 0.1, streamlines in all four cases move downstream of the channel with negligible path crossing (Figure 4a). Any mixing at low Reynolds numbers is dominated by diffusion because crossing of streams does not occur. Some crossing is observed at a Reynolds number of 20 (Figure 4b) and becomes dominant when the Reynolds number is increased to 50 (Figure 4c). Secondary flows start developing with the increase in the flow rate, thereby promoting fluid mixing. The mixing of streamlines also depends on channel geometry, as evident from the crossing of streamlines in different geometries at the same Reynolds number. This is because an increase in curvature also causes a rise in the Dean number. High mixing can be observed in the serpentine channel with sinusoidal walls compared to the simple serpentine channel because of more secondary flow, which becomes prominent as the amplitude of side walls increased. Flow separation also starts with the increase in the fluid velocity and amplitude of side walls. This phenomenon can be observed by the development of separation vortices, which are most effective in the sinusoidal walled channel with *A*/*λ* ratio of 0.2 (Figure 4c(iv)). This increased effect of secondary flows with the increase in curvature is consistent with previously reported results on curvilinear channels [22,23,25].

**Figure 5:**
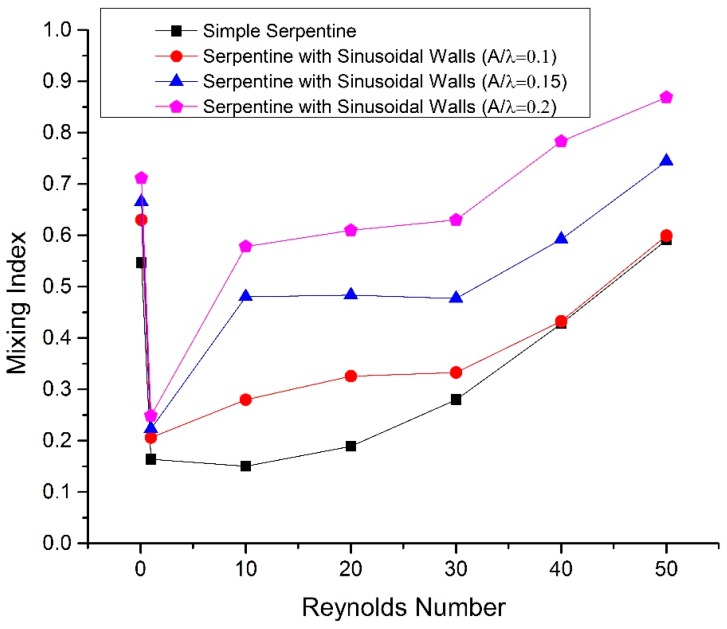
Mixing index vs Reynolds number at the exit.

The mixing index variation with the Reynolds number for all geometries is shown in Figure 5. The mixing index is high at Reynolds number of 0.1 despite strictly laminar flow and the absence of streamlines crossing because, at low flow rate, a long residence time of fluids in the channel allows more time for diffusion to occur, which is the dominant factor that causes mixing in this case. At a Reynolds number of 1, the mixing index sharply declines because the residence time decreases with increasing velocity and is insufficient for diffusion. From Reynolds numbers 10 to 30, the mixing index is steady and does not increase with rise in flow rate because secondary flows are in the development stage and are not fully effective in enhancing the mixing of fluids. From Reynolds numbers 1 to 10, the largest increase in the mixing index is observed for sinusoidal walled channels with *A*/*λ* = 0.15 and 0.2 because of secondary flows, that are not fully developed yet, but are still more effective than the other two geometries because of the high amplitude of the sinusoidal walls that would result in a higher Dean number. The dominant role of secondary flows is evident at Reynolds numbers higher than 30, which increase the mixing index. The mixing index trend with the increase in the Reynolds number for the simple serpentine channel is similar to the previously reported results on simple serpentine geometry [21,29]. Here it is also important to note that the use of sinusoidal walls also increased the total length of the sinusoidal walled serpentine channel compared to the simple serpentine channel. An equal length of both geometries will cause a difference in serpentine shape. Comparison of both geometric shapes with the same total length and the same parameters of serpentine-shaped waves at the same time is not possible. The effect of increased length on mixing performance will be minimal and the enhanced mixing performance of the sinusoidal walled channel is due to the increase in Dean number and secondary flows as explained previously.

**Figure 6:**
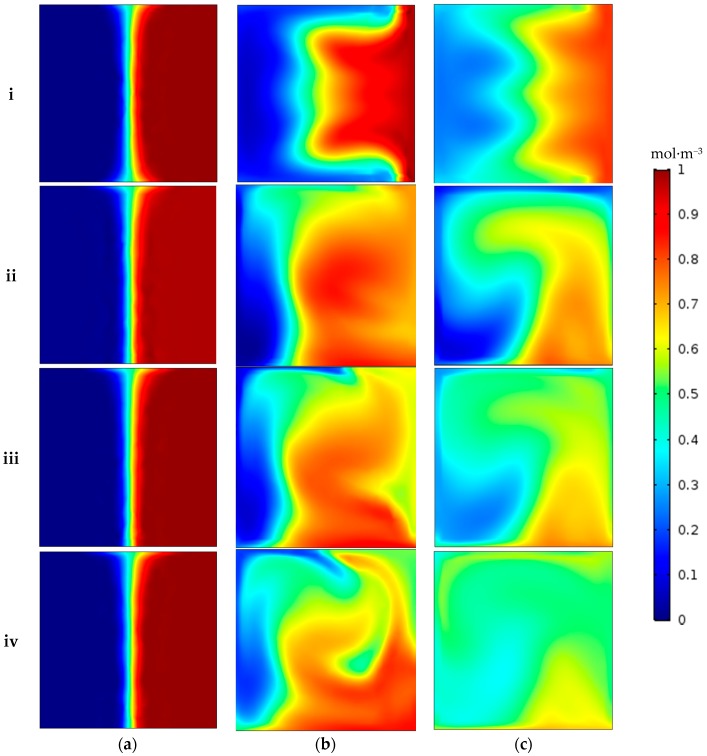
Concentration contours at Reynolds number of 50 at three planes (shown in Figure 1): (**a**) A–A; (**b**) B–B; and (**c**) C–C; for (**i**) Case 1; (**ii**) Case 2; (**iii**) Case 3; and (**iv**) Case 4.

**Figure 7:**
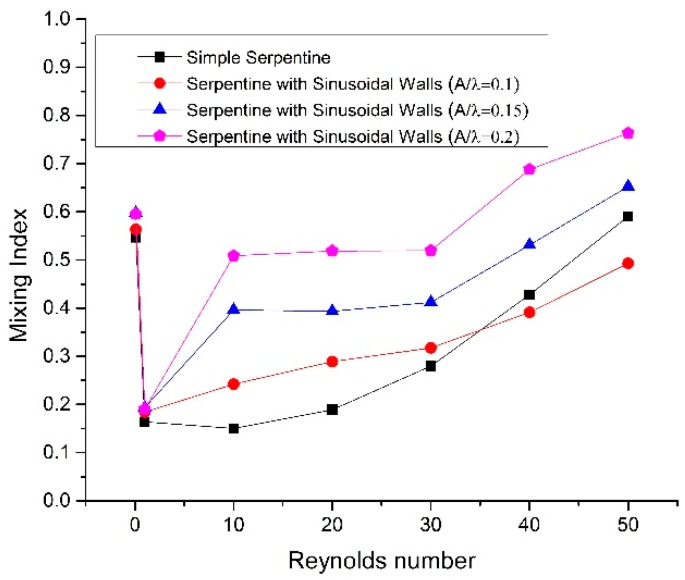
Mixing Index at stream wise length of 6.2 mm.

The concentration distribution of two species at three locations (i.e., planes A–A, B–B, and C–C shown in Figure 1) in all of the geometries at a Reynolds number of 50 is shown in Figure 6. Figure 6a exhibits that both fluid streams are parallel before entering the serpentine region in all geometries with negligible mixing. As fluids move downstream of the channel, secondary flows start developing in the transverse direction, because of turns in the serpentine, and sinusoidal-shaped side walls, thereby enhancing mixing. The concentration distribution becomes more uniform, showing more mixing as the geometric shape changes from simple to sinusoidal walled serpentine and approaches optimal mixing fraction of 0.5 in major portions of the cross section for the maximum amplitude of sinusoidal walls (Figure 6c(iv)). To compare the mixing performance at same total length, the mixing index for fixed stream wise length of 6.2 mm is shown in Figure 7. The mixing trend with same total length for all cases is similar to the mixing trend with the same number of serpentine units but a different total length. The difference occurred at Reynolds numbers after 30 where simple serpentine channel achieved higher mixing index than a sinusoidal walled channel with *A*/*λ* ratio of 0.1. This is because the sharp turns of a simple serpentine caused more secondary flow than the curved turns of the sinusoidal walled channel with low amplitude as illustrated in Figure 4c. This effect is dominated by the high amplitude of sinusoids; thus, the two other cases of sinusoidal walled channels demonstrated better performance.

**Figure 8:**
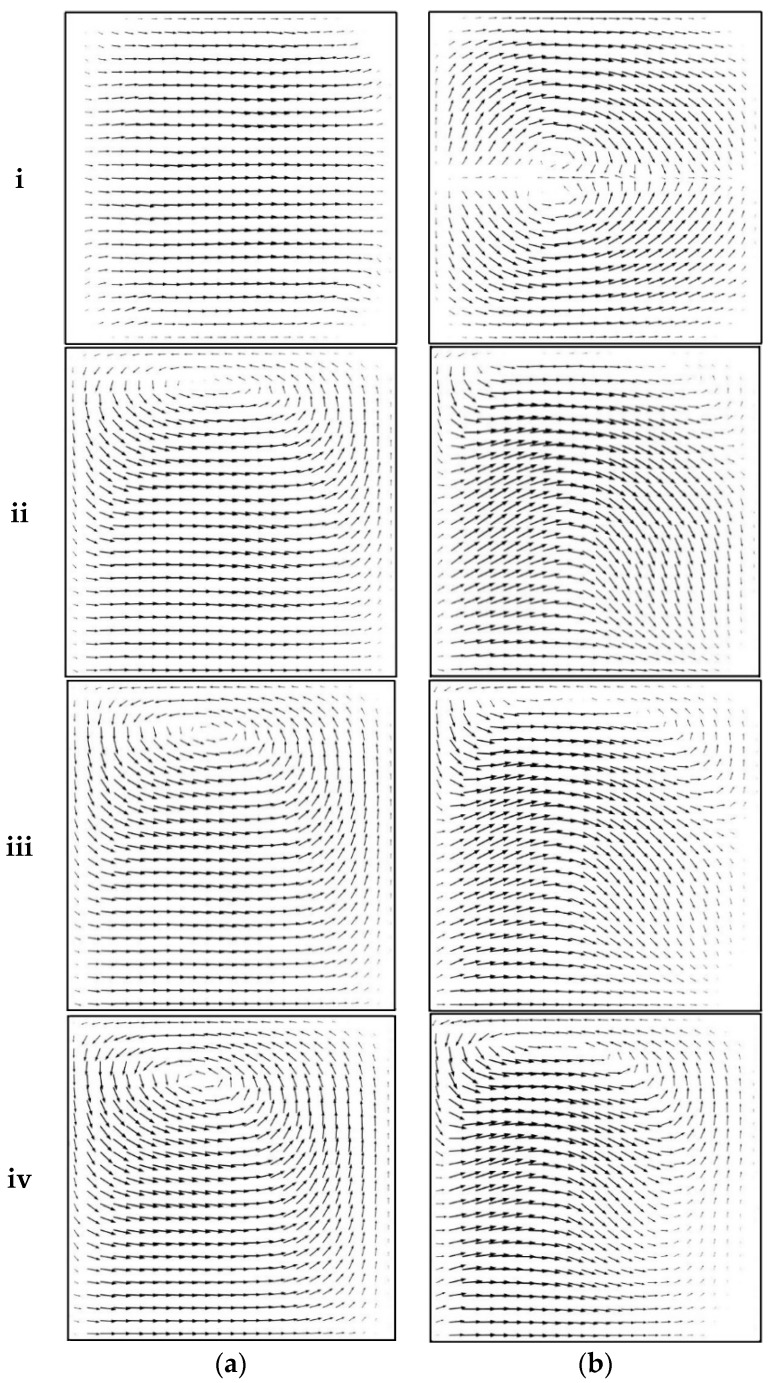
Velocity arrow plots at Plane B-B for all geometries: (**i**) Case 1; (**ii**) Case 2; (**iii**) Case 3; and (**iv**) Case 4 at (**a**) *Re* = 20 and (**b**) *Re* = 50.

Velocity arrow plots to show the secondary flow in the transverse direction at Reynolds numbers of 20 and 50 are shown in Figure 8. The cross section showing arrow plots is located at the Plane B–B shown in Figure 1. No secondary flow is observed in the simple serpentine channel at Reynolds number 20; thus, less mixing enhancement is expected, which corroborates the results shown in Figure 5. For the same geometry, two counter rotating vortices appeared in the central region of the cross section at Reynolds number 50, which explain better mixing for this case. For sinusoidal walled serpentine channels, one vortex with its axis near the side wall appears at Reynolds number 20, as shown in Figure 8a. The axis of the vortex moves toward the central region as the amplitude increased. At a Reynolds number of 50, secondary flow is more prominent in the sinusoidal walled channels with two overlapping counter vortical flows, as shown in Figure 8b. The overlapped area spreads across the cross section with the increase in amplitude; thus, a high mixing efficiency of 86.9% is achieved by sinusoidal walled serpentine channel at Reynolds number of 50.

**Figure 9:**
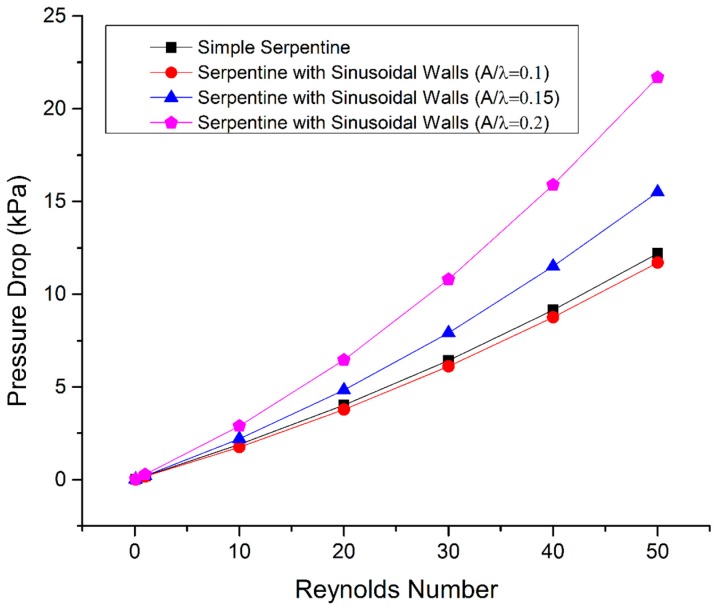
Pressure drop variation with the Reynolds number.

Figure 9 shows the pressure drop across all channels, which is proportional to the flow rate and increases with the increase in Reynolds number. Given that the pressure at the outlet is zero, the pressure drop shows the pumping pressure required at the inlet to drive the fluids through the channel. The sinusoidal walled channel with maximum amplitude has the highest pressure drop, followed by the channel with *A*/*λ* ratio of 0.15 because sinusoidal walls with high amplitude are more resistant to flow than the other cases. The simple serpentine channel has a slightly higher pressure drop than the sinusoidal walled serpentine channel with *A*/*λ* ratio of 0.1 because of a lower resistance to flow from the sinusoidal walls with small amplitude compared to the sharp turns of the simple serpentine channel.

**Figure 10:**
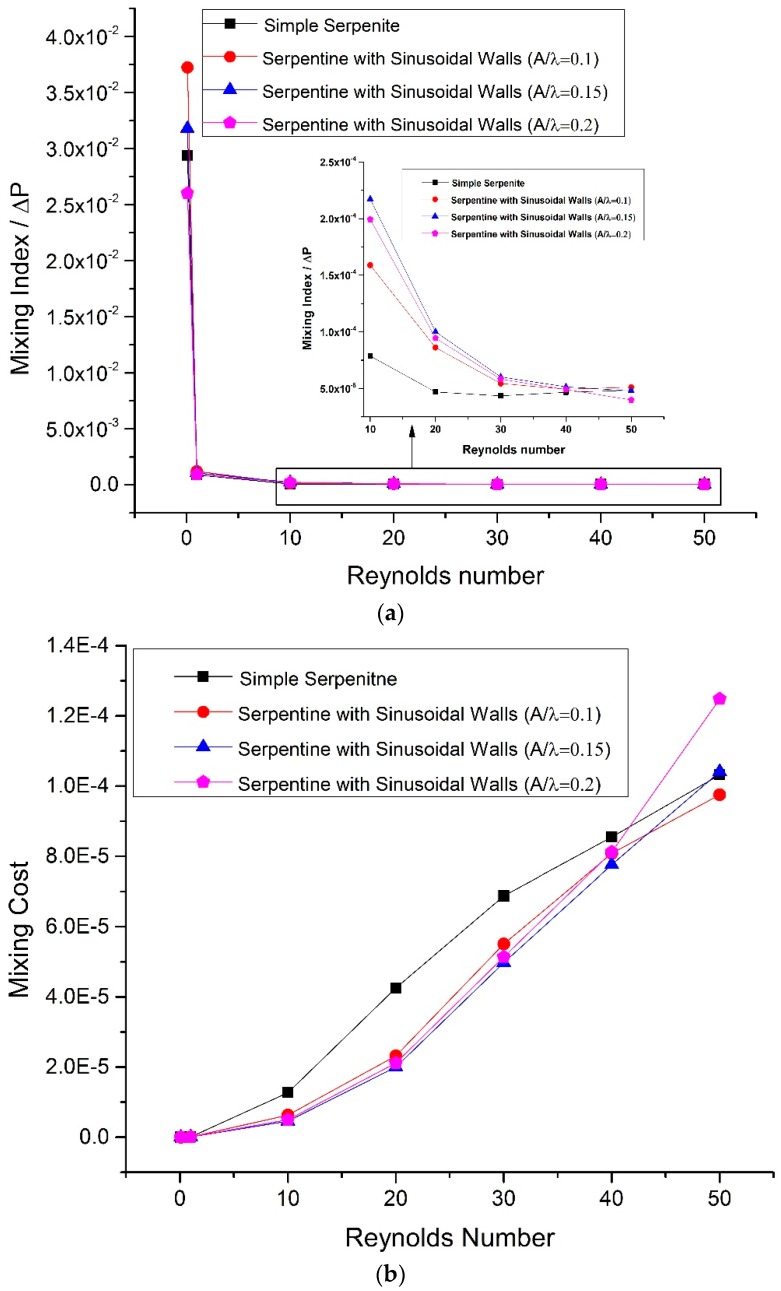
Mixing cost in terms of (**a**) pressure drop and (**b**) input power.

The increase in pumping pressure results in an increased mixing cost because of higher energy consumption. Figure 10a,b show the mixing cost variation with Reynolds number in terms of pressure drop and input power, respectively. A high value of MI/ΔP ratio represents low mixing cost and better performance. At a Reynolds number of 0.1 mixing cost is low because of diffusion dominated mixing with minimum pressure drop but this is not favorable because of the slow processing time. Rapid mixing can be achieved at high flow rates that cause secondary flows. The increase in flow rate also results in increased pressure drop and thus a rise in energy consumption rate is inevitable. So, for any design, mixing cost will always go up with increased flow rate. High MI/Δ*P* ratios of sinusoidal walled channels in Figure 10a show relatively improved mixing performance compared to simple serpentine channels. In the case of mixing cost in terms of input power, a low value represents better mixing performance. The comparison of all cases show that the mixing cost for the three sinusoidal walled cases is similar and comparatively less than the simple serpentine channel except for Reynolds number 50, where geometry with *A*/*λ* ratio of 0.2 achieved a high mixing index at a relatively higher mixing cost (Figure 10b). Below a Reynolds number of 40, the mixing cost of sinusoidal walled channels is relatively lower than the simple serpentine channel.

To characterize rapid mixing, the time required to attain full mixing should also be considered. Although full mixing was not achieved for the number of serpentine waves used in the present study, a comparison based on the mixing index to residence time ratio at different Reynolds numbers is presented in Table 1. A higher mixing index to residence time ratio shows good performance. The residence time was calculated using the length of the serpentine part and average velocity through the channel. Sinusoidal walled channels showed better performance except for a Reynolds number of 40, where simple serpentine showed better performance than sinusoidal walled serpentine with *A/λ* ratio of 0.1 because of sharp turns.

## 7. Conclusions

A chaotic-advection-based serpentine-shaped passive micromixer with sinusoidal side walls was presented in this study, and its mixing performance was evaluated. Numerical simulations were conducted using the Navier–Stokes equations to solve the fluid flow. The resulting velocity field was used to compute the species concentration field using the convection–diffusion equation. The effect of the *A*/*λ* ratio of sinusoidal walls on the mixing efficiency of microchannels was also considered. The mixing performance of the proposed sinusoidal walled serpentine design was compared with a simple serpentine channel at Reynolds numbers ranging from 0.1 to 50. At low Reynolds numbers of 0.1 and 1, mixing occurred because of molecular diffusion and depends on the residence time of fluids in the device. Secondary flows started developing with the increase in Reynolds number and showed more effect in the sinusoidal walled serpentine channels. The dominant effect of secondary flow on mixing was observed at Reynolds numbers higher than 30 in all of the geometries. Apart from the increase in flow rate, the increase in *A*/*λ* ratio of sinusoidal walls also contributed to the growth of secondary flows because of increase in curvature. The proposed design of serpentine channel with sinusoidal walls achieved a comparatively higher mixing index than the simple serpentine channel with a relatively lower or similar mixing cost.

## Figures and Tables

**Table 1:** Comparison of mixing index to residence time ratio.

Geometry	Mixing Index/Time (s^−1^)		
	*Re* = 1	*Re* = 20	*Re* = 40
Simple serpentine	0.2984	6.87	31.12
Sinusoidal walled (*A*/*λ* = 0.1)	0.3380	10.70	28.43
Sinusoidal walled (*A*/*λ* = 0.15)	0.3395	14.71	36.06
Sinusoidal walled (*A*/*λ* = 0.2)	0.3495	17.11	43.98
